# Secure malicious node detection in flying ad-hoc networks using enhanced AODV algorithm

**DOI:** 10.1038/s41598-024-57480-6

**Published:** 2024-04-03

**Authors:** V. Chandrasekar, V. Shanmugavalli, T. R. Mahesh, R. Shashikumar, Naiwrita Borah, V. Vinoth Kumar, Suresh Guluwadi

**Affiliations:** 1 Department of Computer Science & Engineering, Faculty of Engineering and Technology, JAIN (Deemed-to-be University), Bengaluru, 562112 India; 2 Department of Computer Science and Engineering, KSR College of Engineering, Tiruchengode, India; 3 School of Electronics and Communication Engineering, REVA University, Bengaluru, India; 4 Department of Computer Science Engineering, Presidency University, Bengaluru, India; 5 School of Computer Science Engineering & Information Systems (SCORE), Vellore Institute of Technology (VIT), Vellore, India; 6 Adama Science and Technology University, 302120 Adama, Ethiopia

**Keywords:** Flying ad-hoc network, Malicious node, Secure AODV, TAODV, AODV, Computational science, Computer science

## Abstract

In wireless networking, the security of flying ad hoc networks (FANETs) is a major issue, and the use of drones is growing every day. A distributed network is created by a drone network in which nodes can enter and exit the network at any time. Because malicious nodes generate bogus identifiers, FANET is unstable. In this research study, we proposed a threat detection method for detecting malicious nodes in the network. The proposed method is found to be most effective compared to other methods. Malicious nodes fill the network with false information, thereby reducing network performance. The secure ad hoc on-demand distance vector (AODV) that has been suggested algorithm is used for detecting and isolating a malicious node in FANET. In addition, because temporary flying nodes are vulnerable to attacks, trust models based on direct or indirect reliability similar to trusted neighbors have been incorporated to overcome the vulnerability of malicious/selfish harassment. A node belonging to the malicious node class is disconnected from the network and is not used to forward or forward another message. The FANET security performance is measured by throughput, packet loss and routing overhead with the conventional algorithms of AODV (TAODV) and reliable AODV secure AODV power consumption decreased by 16.5%, efficiency increased by 7.4%, and packet delivery rate decreased by 9.1% when compared to the second ranking method. Reduced packet losses and routing expenses by 9.4%. In general, the results demonstrate that, in terms of energy consumption, throughput, delivered packet rate, the number of lost packets, and routing overhead, the proposed secure AODV algorithm performs better than the most recent, cutting-edge algorithms.

## Introduction

**Figure 1:**
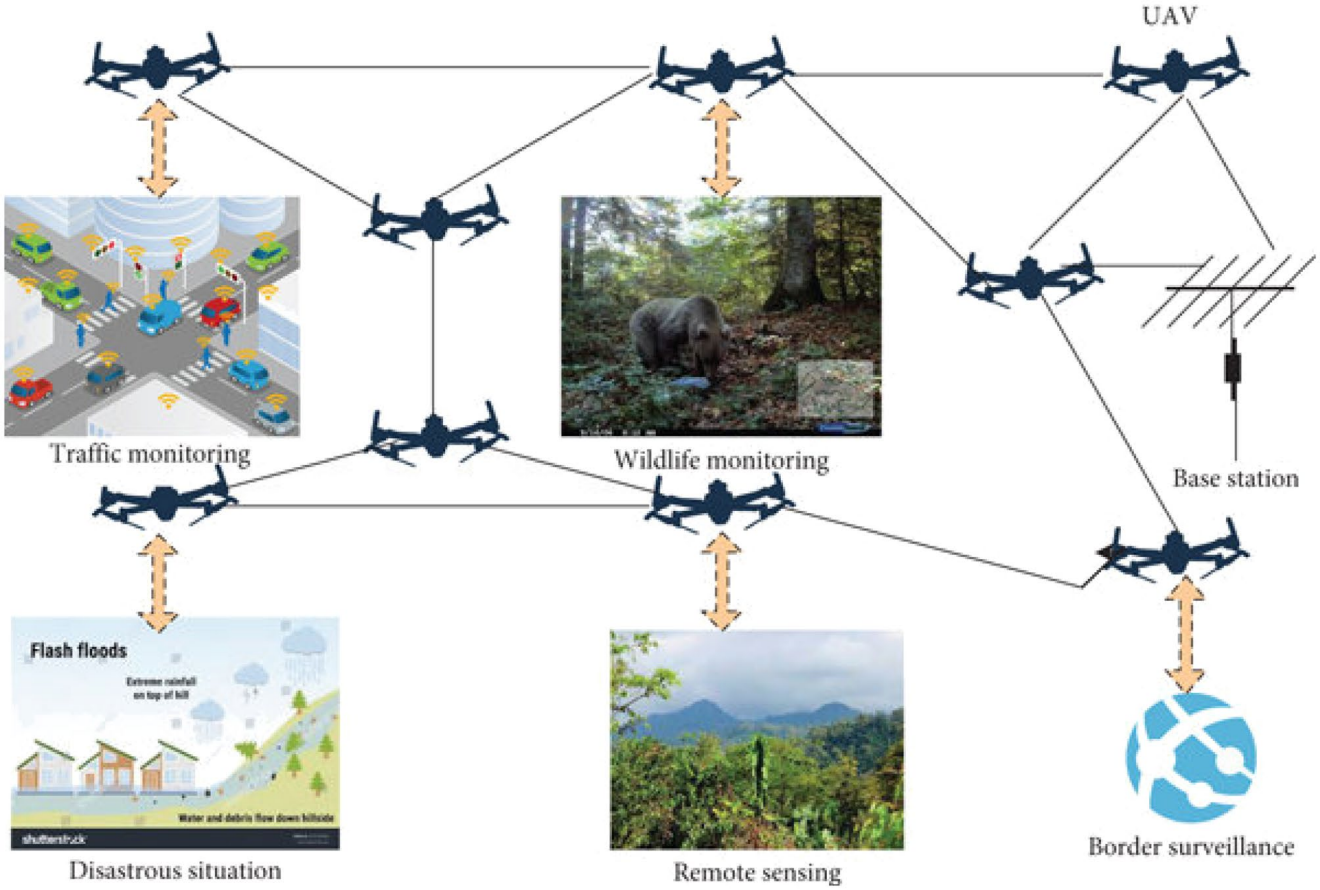
FANET model.

A flying ad hoc network (FANET) refers to a specialized drone network utilized for transmitting gathered data to ground stations within diverse environments. FANET employs various aerial vehicles such as drones, small aircraft, and balloons. These are pre-programmed remote controls and networks^1^. Web applications for unmanned aerial vehicles are used in emergencies such as floods and civilian applications. The use of FANET for various military applications for the general public and businesses is reliable and important for those who transmit mobile -related information to get great results. Motion confirmation, remote sensing, ambient surveillance, fault management, social insurance observation and switching systems are some of the areas where FANET is used^2^. The FANET model is shown in Fig. 1.

For example, in reconnaissance applications such as perimeter surveillance frames and city observations, drones play a key role in limiting intervention. Drones can screen and report information about incidents and manage activity are an economic and socially economical option because they do not have to travel long distances. We can also exchange special information for situations important for drones to travel at high speeds, and support investigations and rescue operations^3^. The remote sensor system (WSN) and UAV system can be used to protect territories such as forests and swamps that are inaccessible to ground vehicles due to remote areas from uncontrollable fire damage. Because of unresolved security issues related to FANET, limited bandwidth, malicious outgoing messages, insecure data transmission, dynamic channel creation, dialing, unmanned communication, and routing^4^. FANET is a special type of MANET with high mobility. However, despite its many advantages, FANETS is specifically designed and carries challenges associated with communications. For optimal operation, each FANET node must have expensive hardware capable of communicating with terrestrial nodes or satellites. Another problem of FANET is the reliability of communication. Safety is the biggest challenge of the FANET. Various attacks are happening on the FANET. These attacks occur when rogue nodes are damaged in your network. Network security is defined as the retention of personal information and identity verification^5^. Another major problem faced by such networks is the communication between ground drones, which ensures confidentiality, and availability. In a network where legitimate nodes act like rogue nodes, different types of attacks will occur, such as Nashville attacks, black holes, wormholes, and cyber-attacks. An acoustic communication encounters challenges like limited bandwidth and extended propagation delay. Conventional methods for optimizing localization suffer from drawbacks such as increased energy consumption and costs^6^. To overcome these obstacles, we introduce a novel hybrid optimized localization technique specifically designed for underwater sensor networks (UWSNs), facilitating precise localization of mobile nodes. This approach integrates various factors into a comprehensive fitness function, encompassing metrics like the number of hops from the anchor node, time of arrival (ToA) distance estimation error, and transmission delay. Nonetheless, current protocols may struggle to deliver accurate data in vast aquatic environments such as seas and oceans due to their extensive coverage^7^. Moreover, UWSNs confront additional hurdles, including fluctuations in water level and pressure, demanding thorough examination. In response, sensor network designers advocate for the utilization of machine learning methodologies and relevant pseudo code to craft effective prediction models capable of tackling these intricacies. The difference between UWSN and FANET is summarized in Table 1.

**Table 1:** Difference between UWSN and FANET.

Study	Year	Methodology	Findings	Remarks
Nain et al.^6^	2022	Node localization scheme for UWSNs	Energy consumption, localization error, delay and cost	It often exhibit increased latency and error, even with a large deployment of anchor nodes
Goyal et al.^7^	2019	Data agglomeration in UWSNs	Routing using SOM and reinforcement learning	Addresses data accuracy and variability challenges in UWSNs, using machine learning solutions
Study under Consideration	–	Secure data routing for FANET	AODV protocol and trust computation model	To solve both gaps in^6,7^ and make the finding to ensure the security of FANET

## Research motivation

An attacker exploiting this vulnerability could potentially gain access to network processes, posing a significant threat to FANET security, which is paramount to overall network security. However, despite its numerous advantages, FANET also presents challenges such as resource constraints, portability requirements, open communication channels, and shifting organizational geographies. Particularly in critical areas like military operations, disaster management, and border surveillance, FANET technology is indispensable. Yet, its high mobility and decentralized nature make it susceptible to various security threats, with malicious nodes posing a significant risk by disseminating false information, disrupting routing, or launching attacks against legitimate nodes, thereby jeopardizing network operations. Traditional security measures like encryption and access control are inadequate for the dynamic nature of FANETs, necessitating the development of rapid and reliable methods for identifying and eliminating malicious nodes from the network. In this context, our work aims to devise a threat detection strategy capable of pinpointing and isolating malicious nodes in FANETs, thereby bolstering the network's security and reliability. The proposed secure AODV algorithm plays a crucial role in this effort, effectively detecting and isolating malicious nodes to purge false information from the network. The contributions of our work can be summarized as follows:

1. Incorporating trust models based on direct or indirect reliability adds an extra layer of defense against attacks on vulnerable temporary flying nodes.
2. The proposed method enhances FANET security performance by reducing packet loss and routing overhead while simultaneously increasing throughput compared to conventional algorithms like TAODV and AODV.
3. By employing our proposed technique for threat detection in FANET, the early identification and prevention of security breaches in real-time can be significantly improved, thus enhancing the reliability and security of FANET operations.

The hypothesis of proposed contributions is given as follows:

- By incorporating trust models based on direct or indirect reliability and implementing a secure AODV algorithm, we hypothesize that improve the security performance of FANETs, reducing packet loss and routing overhead while increasing throughput compared to conventional algorithms.
- We expect that our proposed technique for threat detection will significantly enhance the early detection and prevention of security breaches in real-time, thereby making FANETs more reliable and secure.

## Literature survey

In this part, we give an outline of existing examination and studies connected with the location of vindictive hubs in flying specially appointed networks. The section begins with an introduction to FANET and its security challenges, followed by a discussion of the various approaches used to detect and isolate malicious nodes.

### Security challenges in FANET

UAVs have few features like high portability, frequent structural changes and three-dimensional movement in space and it was difficult to design a novel routing protocol for the FANET. In this case, we believe that topology -based routing is the most important technique to solve the FANET routing problem^8^. The OPNET (optimized network engineering tool) simulator was also used to evaluate based on the end-to-end latency, and throughput. FANET has become an alternative access technology for hard-to-find areas where there is no fixed infrastructure. An unmanned aerial vehicle (UAVs) communicates with each other and does not have routing protocol^9^. This paper proposed an adaptive FANET routing protocol based on a cleaning system to use Network Simulator 2 (NS-2) for simulation and quality of service (QoS) to validate the adaptive FANET protocol, and evaluate it based on the quality of the experience. With the emergence of the Ad Hoc Flying Network (FANET) created new value-added services^10^. The proposed mechanism is based on the central gravity positioning technique, which uses fuzzy logic to calculate the position of the unknown node of the UAV^11^. UAV nodes are made up of clusters. To reduce power consumption and extend the lifetime of the network, the proposed positioning algorithm selects the channel head and drone first^12^.

Peer-to-peer networks are a group of wireless mobile hosts that form peer-to-peer networks without the help of an integrated infrastructure or centralized management. In this environment, the limited wireless transmission range of each mobile host requires the mobile host to send data packets to the destination using other hosts^13^. This article describes an ad hoc network routing protection that uses dynamic resource routing. When the host moves frequently, the protocol can quickly adapt to routing changes, but the time required moving the host frequently requires almost no overhead. One of the most important aspects of implementing FANET in a security sensitive environment is very challenging. Compared to wired networks, the FANET is a security hole because it lacks centralized trusted privileges^14^. In addition, because temporary flying nodes are vulnerable to attacks, trust models based on direct or indirect reliability similar to trusted neighbors have been incorporated to overcome the vulnerability of malicious/selfish node attack^15^. An ad hoc network between drones solves problems that arise in network -based infrastructure. To analyze the self-organizing flying network (FANET) that connects drones. First, the distinction between FANET, MANET and VANET is clarified, and the main FANET design issues will be introduced^16,17^. It is released along with the existing FANET protocol and also solves research problems. The new model of trust and host selection for ad hoc mobile content delivery networks used to analyze and evaluate this model can be compared to this model^18^. The presented results show that simply selecting the best node model can lead to serious imbalances associated with node workload and the probabilistic model can defeat this problem, and at the same time can download a lot of useful content from reliable and properly selected nodes. Due to the high speed of navigation, environmental conditions and soil structure, this special mobile routing protocol is not suitable for FANET^19^. The proposed method provides a mixed use of unicast routing and geocoding using location and orbit information. The FANET and VANET security architecture aims to detect and isolate malicious nodes, additionally, it directs the search for the bogus identities in the self-organized network system become unstable and network security will be reduced^20^.

### Secure aware routing algorithm for FANET

A FANET classification method called belief-based clustering scheme (TBCS)^21^ uses the Takagi–Sugeno-Kang fuzzy inference method and the multi-criteria fuzzy method to examine the behavior of nodes in an environment that is complex and uncertain. To differentiate between malicious and non-compliant FANET nodes and evaluate node behavior, TBCS uses a reward and punishment system. A dependable cluster head is chosen to carry out inter-cluster and ground control station communication based on the calculated reliability values. GPS-spoofing attack conspirators are identified by FANET systems using a policy-based distributed detection mechanism^22,23^. Witnesses are used to verify and identify GPS jamming signals and are categorized as active or passive based on their location at the time of the attack. Signals are identified by measuring the carrier-to-noise ratio and absolute power. A conviction model that limits misleading bits of hearsay and coordinates various sources is executed utilizing beta and Weibull disseminations^24,25^. As indicated by reproduction results, GPS parody signs can be identified and dropped with almost 100% high exactness and low correspondence cost. In order to ensure energy-efficient and secure optimal routing in a FANET, a WOA-OLSR algorithm based on the wavelet optimization algorithm is proposed^26^. The best exhibition of this calculation was assessed utilizing different execution boundaries, for example, bundle conveyance rate, delay, energy utilization, throughput and intricacy. This demonstrates that it improves OLSR's performance. SEEDRP is a dynamic method for driving energy-efficient and safe vehicles. Two steps are involved: security and SEEDRP-based routing^27^ Using a dynamic routing algorithm, the best route from the source node to the destination node is determined in the first step. The final step is a dynamic key generation scheme for safe data transfer. The findings demonstrate that SEEDRP boosts energy efficiency and quality of service without affecting network performance. In order to improve network performance, the reliable link adaptive position-based routing protocol (RLPR)^28^ has been proposed for FANET. Using a forwarding angle, it takes advantage of the nodes' relative speeds, signal strength, and energy, as well as the geographical distance to the destination. Based on signal strength and relative speed, RLPR selects relay nodes in the forwarding area moving in the target direction^29^. It uses more energy to choose the next hop and achieves a higher connection state. Control message overhead, network lifetime (by 55% and 65%, respectively, compared to RARP and AODV), and search success rate (by 26% and 28%, respectively, compared to RARP and AODV) are all significantly enhanced by RLPR. The research gaps in the current FANET secure routing algorithms are summarized in Table 2.

**Table 2:** Summary of research gaps.

References	Year	Technique	Algorithm used	Outcomes	Research gaps
^21^	2020	Trust based grouping plan for secure correspondence	Takagi–Sugeno–Kang fuzzy inference	Delay, drop rate and loss rate	Network resources like bandwidth, energy, and processing power are typically limited in FANETs
^22^	2021	Identification of conspiratorial GPS-Spoofing attacks	Beta and Weibull Distribution	Attack detection and miss rate	FANETs are defenseless against different security dangers like sticking, snoopping, and caricaturing assaults
^26^	2021	System of communication for energy security and efficiency	Advanced Interface Level Control Utilizing Wavelet Simplification (WOA-OLSR)	Delivery ratio, delay, energy utilization, throughput	It is difficult to maintain a stable and effective routing path between nodes because of their mobility
^27^	2021	Protocol for secure, energy-efficient, dynamic routing	Distinct dynamic key (DDKey)	Delay, drop rate and loss rate	It is challenging to develop routing protocols that can adapt to the network's size for FANETs with multiple nodes
^28^	2021	Dependable connection versatile position-based steering	Fuzzy inference model	Energy, throughput, network lifetime	In order to support applications like video streaming and real-time communication, FANETs require a certain level of quality of service (QoS), such as low latency and high bandwidth

## Proposed methodology

Due to the decentralized and mobility environment of the network, fraudulent nodes will enter the network. These fraudulent hosts initiate various types of active and passive attacks. The legitimate host ID is forged as a result of an active attack called a Sybil attack. As the requested data has not yet reached the legal node, the efficiency of the network is reduced to a minimum. Because there are malicious nodes identified by search and removed using the proposed method, Sybil attacks will occur on the network. Introduce signal strength in the proposed method based on the tracking model technique. Using the proposed technology, internet control message protocol (ICMP) messages are propagated from roadside equipment to the network. Collect complete information one by one and share data. Suppose a node can break into a network with multiple signal strength values. Check the packet, and then send it through the network one by one to see which node is malicious. Entered monitoring mode, received control data packets, and began observing vehicles at nearby nodes. Here, we apply multi-path routing to identify malicious nodes and remove malicious nodes in the network.

The basic steps to solve security issues in FANETs which are given as follows:

(i) Trust mechanism
(ii) Threats detection

Trust mechanism is a technique used to assess the reliability and credibility of nodes in a network based on their past behavior, interactions, and other relevant factors. Trust mechanisms can be used to improve network security by identifying malicious nodes and preventing them from accessing the network or performing harmful actions. Threat detection is the process of identifying and responding to potential security threats in a network. Monitoring network traffic, examining system logs, and utilizing machine learning algorithms to identify anomalies or suspicious behavior are all examples of this. The identification and mitigation of security threats in advance of their potential harm to the network or its users is the objective of threat detection.

### Trust mechanism

The proposed method must determine the reliability of nodes in the network. Due to the wireless communication between FANET nodes, there is always a risk that the data will be destroyed by unauthorized nodes^21^. Those nodes are usually outside the network, but over time, it is likely that many nodes in the network will be damaged or gradually destroyed. Detect and remove malicious nodes and protect the network from damaged internal nodes and external attacks. FANET nodes move very fast, so the reliability value must be calculated, verified and updated very quickly. Neighboring hosts assign a default trust value of 0.5 to all hosts that model the network. The range of reliability values ​​is from 0 to 1. The confidence limit is set to 0. A node with a confidence value of 0 for the specified threshold is treated as a trusted or benign node. A node with a reliability value of 0 or a value of 0 will immediately be marked as a malicious node and removed from the network. When the reliability value increases by 0.001, the packet is sent to the destination node. When a node is not packed, the reliability value of the node will decrease to 0.001, as it requires more transmissions. Data is transferred/forwarded only to trusted nodes. The trust table is updated periodically to maintain control over all nodes in the network. The confidence level is determined by the location of the most popular FANET hub in the search area and its proximity to it as shown in Eqs. 1 and 2.

1 $$Y_{j} (s + 1) = Y_{j} (s) + v_{j} (s + 1)$$

2 $$v_{j} (s + 1) = \omega \times v_{j} (s) + C_{1} R_{1} (Y_{qbest} - Y_{j} (s) + C_{2} R_{2} (Y_{hbest} - Y_{j} (s))$$

where and any number between 0 and 1 are positive acceleration coefficients. To determine how the previous speed affects the current speed, the inertia weight w is used. This particle's best position is hQest, and the best position of any other particle is hBest. In this case, we employ the quantum to neutral FANET node ratio. Quantum FANET nodes, on the other hand, boost performance by lowering the ratio of quantum to neutral particles when the environment abruptly changes. The number-choice capability of quantum FANET hubs is described as follows as shown in equations from 3 to 5.

3 $$dist_{s} = |hk_{s} - hk_{s - 1} |$$

4 $$Q_{out} = count\{ dist_{s} |dist_{s} > w_{r} ,\,s = 1,\,....,\,S\} /S$$

5 $$n_{P} = (1 - Q_{out} ) \times 0.9$$

where $hy_{s}$ WR is the powerful range of the FANET hubs fixated on the best position, S is the size of the natural dataset that the RBL model is thinking about, and Dist. is the distance that is under 1 from the best situation in age s to the best situation in age s. The momentum technique for website streamlining is recognized $hY_{now}$. We use the database reference of the first K closest data nodes as shown in Eq. 6 when detecting changes. The distance between reference data and reference gravity is $hY_{now}$.

6 $$d_{r} = \max \{ |hy_{j} - hY_{now} |,\,\,\,j = 1,...,\,k\}$$

**Figure 2:**
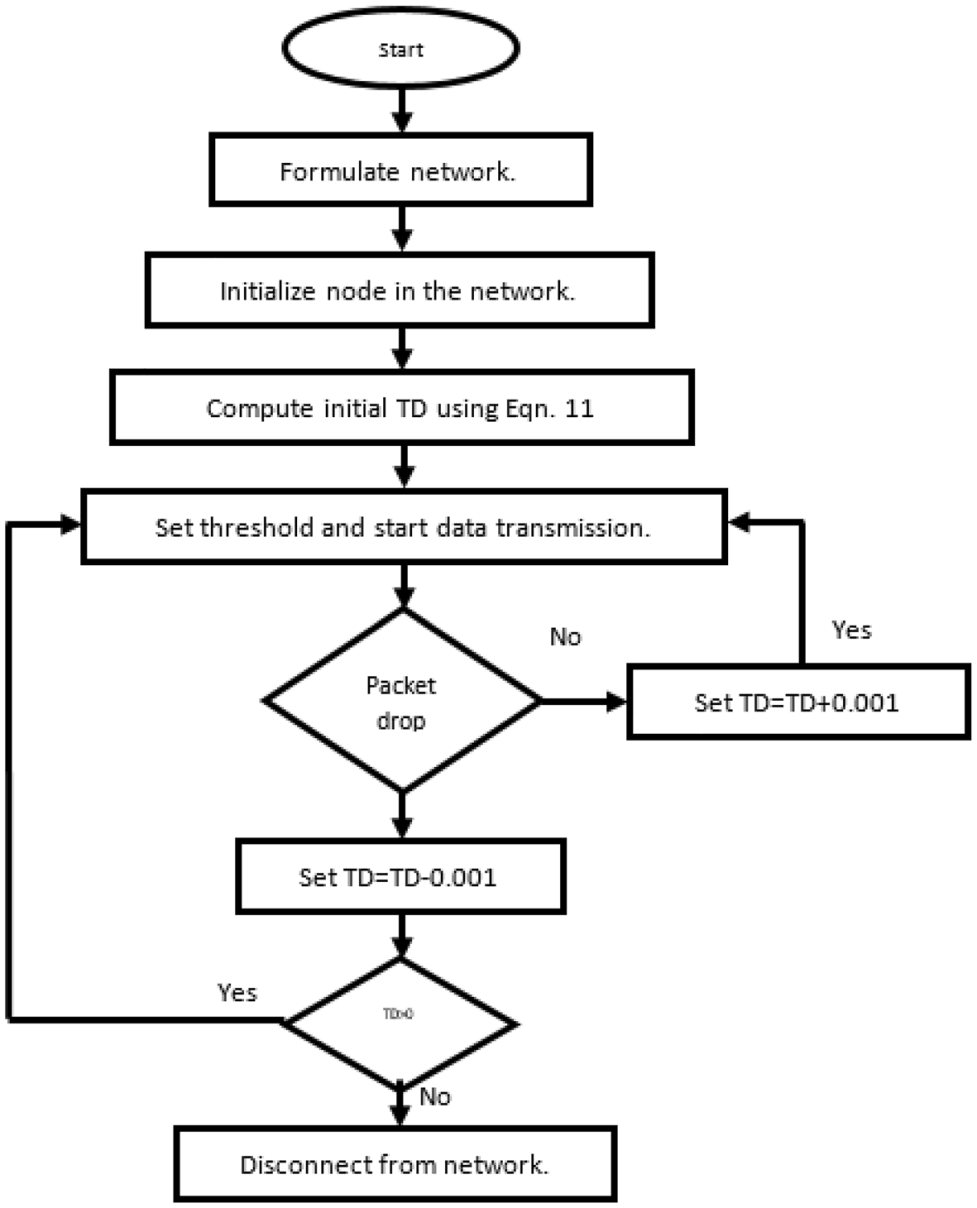
Flow chart of Secure AODV.

When cue difficulty is high, cue reliability is low, indicating a lack of similarity to the situation before. Flow chart of Secure AODV is shown in Fig. 2. The expected data and the anticipated potential region are connected with the help of a least-squares circle. The smallest circum circle's diameter is a measure of predictive difficulty as shown in Eqs. 7 and 8:

7 $$d_{q} = \max \{ |QY_{j} - Q_{i} |,\,\,\,\,j = 1,....,k$$

8 $$n_{P} = (1 - Q_{out} ) \times 0.9$$

When the forecast intensity is high and the forecast data are sparse, the smallest circle is larger. On the other hand, the estimated data converge when the difficulty of the estimation is low. Additionally, we employ the well-known Best Generation (BOG) strategy.

Marsh is a calculation as shown in Eq. 9 that identifies the system with the most important health values and is a measure of optimality. BOG performs multiple runs to determine the best solutions for each iteration.

9 $$AOH = \sum\limits_{j = 1}^{r} {\sum\limits_{i = 1}^{n} {\frac{{fitness_{ji} }}{r * n}} }$$

where n is the number of runs and r is the number of replicates for each experiment. Natural environmental changes are used in our trust model. The value of the next best growth rate is shown in Eq. 10:

10 $$Rate_{OI} = \frac{MQSO - QBLDSO}{{MQTO}}$$

The following is the step-by-step procedure for determining the rate of improvement as shown in Eq. 11:

11 $$Rate_{O2} = \frac{MPSODE - QBLDPSO}{{MPSODE}}$$

When the transition period and confidence level (TD) are at their highest, our confidence model significantly improves as shown in Eq. 12.

12 $$TD = \frac{{Rate_{OI} }}{{Rate_{O2} }}$$

The operational flow of the trust mechanism for the secure AODV algorithm is shown in the Fig. 2.

### Threats detection

To comprehend how nodes enter and exit the UAV network, it's imperative to conduct a thorough analysis of the network and its components. A technique is needed to ensure the consistency of neighbor information and to identify false identities. In this work, we propose a novel approach for detecting malicious network nodes responsible for network attacks. Such attacks can lead to wasted network resources and reduced network throughput, along with increased data packet delay due to packets being incorrectly routed or taking longer paths to reach their destination. The assumptions formulated for this study are as follows:

- The mobility of UAV nodes follows dynamic paths.
- A central controller updates and stores information about UAV nodes.
- UAV nodes must provide information about neighboring nodes to the central controller.
- The central controller has the capability to store information about the neighboring nodes of all nodes in the network.

This research focuses on detecting malicious network hosts responsible for network attacks, particularly distributed denial of service (DDOS) attacks. In a DDOS attack, a malicious node selects a legitimate node to attack the victim's node. During the attack, the malicious node sends control packets to the legitimate node, which then forwards routing data packets to the victim node to launch the attack. The proposed strategy as shown in algorithm 1 for detecting malicious nodes involves the following steps:

- To understand the dynamics of entry and exit within the UAV network, a comprehensive study of the network and its constituent components is necessary. A novel approach based on the detection of malicious network nodes, responsible for instigating network attacks, is proposed in this work. The occurrence of a DDOS attack on the network occurs when a malicious node selects a legitimate node to target the victim's node.
- During the attack, the malicious node sends control packets to the legitimate node, prompting the legitimate node to route data packets to the victim node, thereby initiating the attack. This task proposes a strategy to detect malicious nodes through several steps.
- Firstly, the network is divided into a finite number of nodes, with each node assigned a fixed spare capacity. Bandwidth utilization for each node is then analyzed, with nodes exceeding their allocated bandwidth considered potential malicious nodes.
- Furthermore, the type and frequency of data packets transmitted by each node are examined, allowing for the identification of abnormal packet transmission patterns indicative of malicious behavior.
- Subsequently, victim nodes, targeted by the malicious nodes for attack, are identified based on the received control packets from other nodes. By employing this comprehensive approach, the UAV network can effectively detect and neutralize malicious nodes, bolstering network security and reliability.

**Algorithm 1:**
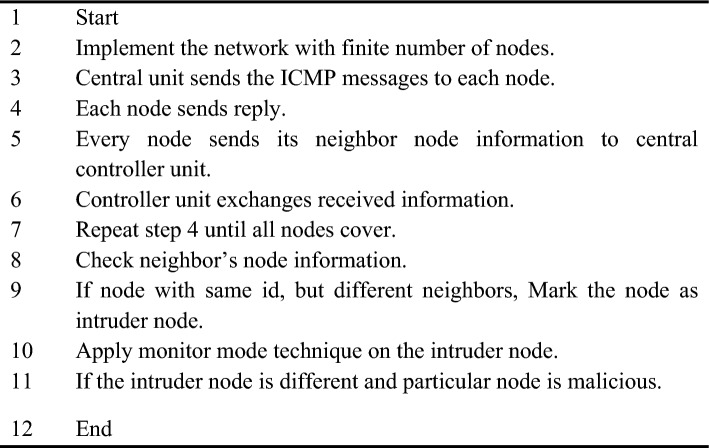
Process of threats detection from FANET.

## Results and discussion

In this section, we assess the effectiveness of our proposed secure AODV algorithm across various simulation scenarios. Utilizing the network simulator-2 (NS2) version 2.35, we implement and evaluate the performance of our algorithm. Comparative analysis is conducted by juxtaposing the simulation outcomes of our proposed secure AODV algorithm against those of established state-of-the-art techniques, including traditional AODV and trusted AODV. Performance is evaluated based on multiple metrics, including energy consumption, throughput, packet delivery ratio, packet loss ratio, and routing overhead.

### Simulation setup

**Figure 3:**
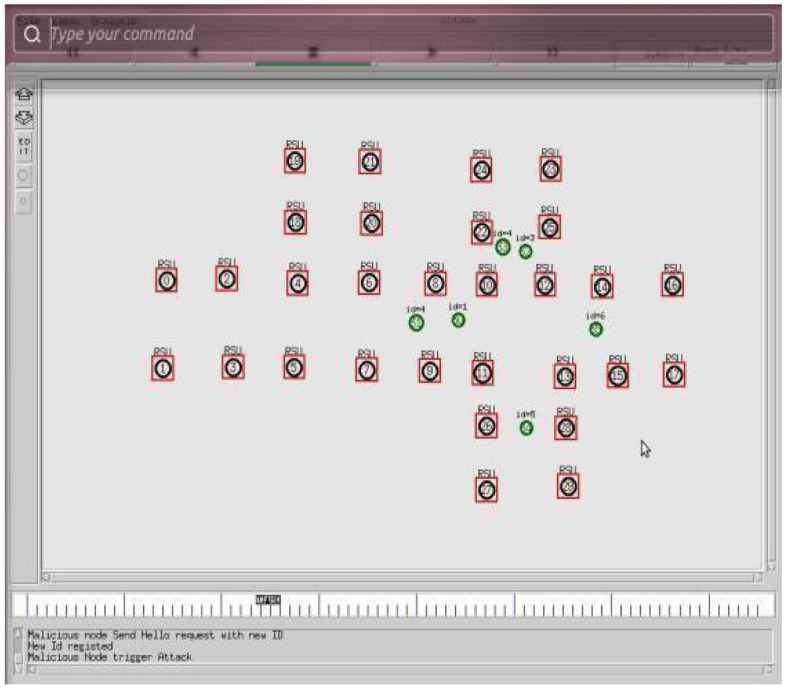
Malicious nodes in FANET.

**Figure 4:**
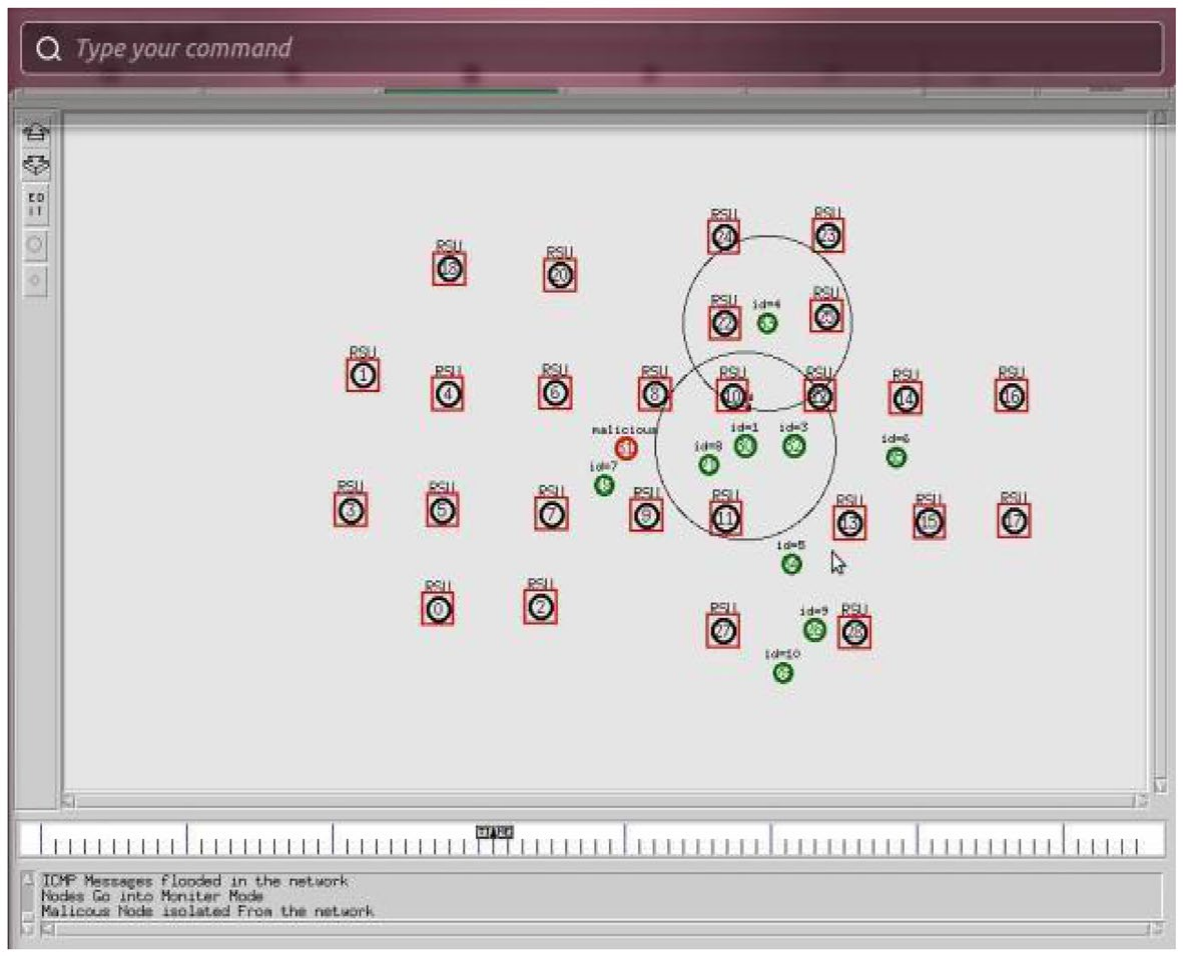
Malicious nodes detection in FANET.

The Table 3 shows the simulation parameters used in the study. The simulator used was NS 2 and the simulation area was set to 1000 × 1000. The number of nodes varied from 20, 40, 60, 80 to 100, while the number of satellites was set to 7. The number of UAVs varied from 5, 10, 15, 20 to 25. The radio propagation model used was free space, and the mobility model was the random waypoint. The node speed was set to 20 m/sec, and the packet size was 512 Kb. The protocol used was secure ad hoc on-demand distance vector (S-AODV), and the packet type was TCP. The simulation time varied from 5, 10, 15, 20 to 25 s with a pause time of 15 s. The number of exchange packets was set to 1000. These parameters were used to evaluate the performance of the proposed S-AODV algorithm for detecting and isolating malicious nodes in FANETs. Figure 3 depicted the central controller unit (CCU) detects that there is a malicious host in the network, the CCU repairs the network. The unmanned node in the network receives a greeting and begins to monitor its neighbors. Use monitor-mode technology to detect malicious hosts on the network. Figure 4 depicted the central manager detects that a malicious node has entered the network, it will take the ICMP message to its neighbors through the network.

**Table 3:** Simulation Parameters.

Parameters	Values
Simulator	NS 2
Simulation area	1000*1000
Number of nodes	20, 40, 60, 80 and 100
Number of Satellites	7
Number of UAVs	5, 10, 15, 20 and 25
Radio propagation model	Free space
Mobility model	Random way point
Node speed	20 m/sec
Packet size	512 Kb
Protocol	S-AODV
Packet type	TCP
Simulation time (pause time)	5, 10, 15, 20 and 15 s
No. of Exchange packets	1000

## Comparative analysis

In this section, the performance of the proposed and existing algorithms is compared in two different scenarios: varying number of nodes and simulation pause time. The aim is to validate the effectiveness of the proposed algorithms and determine how they perform under different conditions. The comparative analysis is conducted using various performance metrics, such as energy consumption, throughput, packet delivery ratio, packet loss ratio and routing overhead. This analysis provides insights into the strengths and weaknesses of each algorithm and helps in identifying the best algorithm for a given scenario.

The Table 4 presents the comparison of the proposed Trust AODV and Secure AODV algorithms with the existing AODV algorithm in terms of the number of the simulation's nodes. In terms of average end-to-end delay, it can be seen that the proposed Trust AODV and Secure AODV algorithms perform better than the existing AODV algorithm. The average end-to-end delay difference between the proposed algorithms and the existing AODV algorithm gets bigger as the number of nodes grows.

**Table 4:** Results comparison of proposed and existing algorithms with respect to number of nodes.

Number of nodes	Energy consumption (J)			Throughput (Mbps)			Packet delivery ratio (%)		
	AODV	Trust AODV	Secure AODV	AODV	Trust AODV	Secure AODV	AODV	Trust AODV	Secure AODV
20	15.030	10.133	5.236	60	80	140	84.486	90.175	95.864
40	16.689	11.792	6.895	70	90	145	83.378	89.067	94.756
60	17.689	12.792	7.895	80	110	150	81.857	87.546	93.235
80	19.252	14.355	9.458	90	140	165	81.634	87.323	93.012
100	22.361	17.464	12.567	105	150	175	79.857	85.546	91.235

Number of nodes	Number of packet loss			Routing overhead (%)					
	AODV	Trust AODV	Secure AODV	AODV	Trust AODV	Secure AODV			
20	130	80	20	15.372	12.804	10.236			
40	150	110	30	17.704	15.136	12.568			
60	170	130	40	23.794	21.226	18.658			
80	180	140	50	26.371	23.803	21.235			
100	195	150	60	27.616	25.048	22.480			

**Figure 5:**
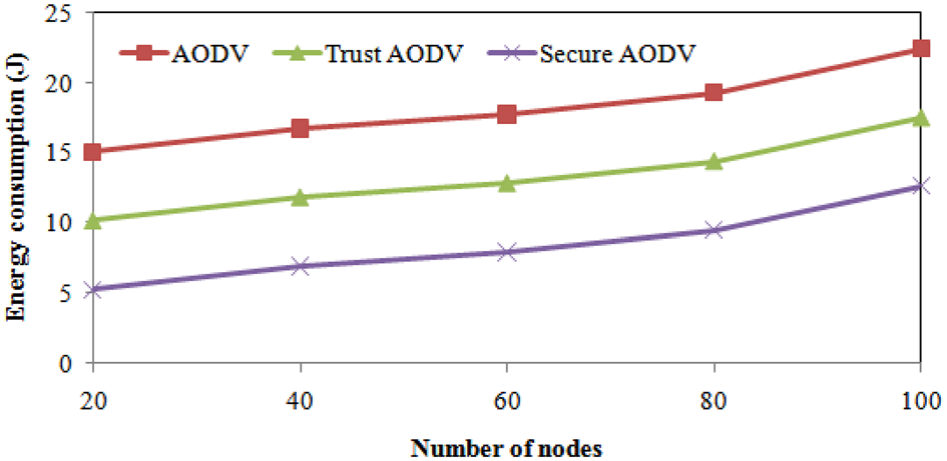
Energy consumption comparisons with number of nodes varying.

**Figure 6:**
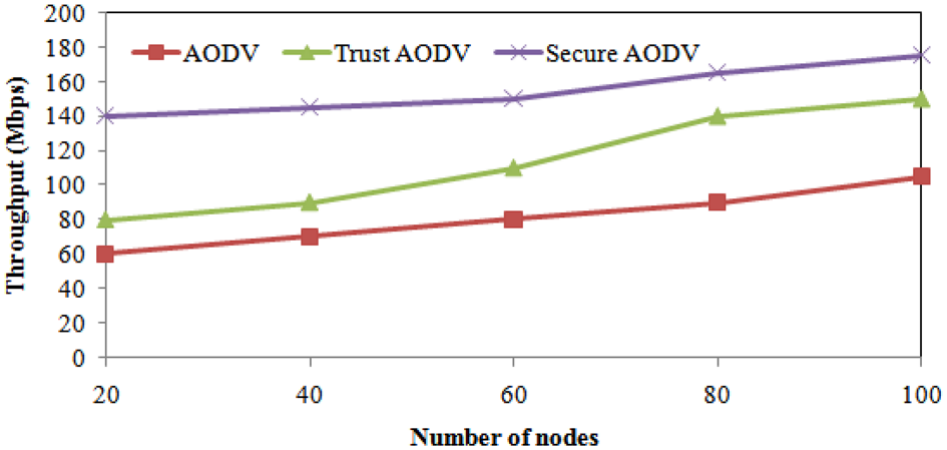
Throughput comparisons with number of nodes varying.

Secure AODV algorithm shows the best performance with the shortest average delay from beginning to end of the three algorithms for all numbers of nodes. Figure 5 shows the energy consumption comparison of proposed and existing algorithm over number of nodes varying. Comparing the energy consumption of the proposed and existing algorithms for different numbers of nodes, we observe that Trust AODV and Secure AODV both have significantly lower energy consumption than AODV. For instance, with 20 nodes, Trust AODV reduces energy consumption by 32.49% compared to AODV, and Secure AODV reduces it by 65.13%. With 100 nodes, Trust AODV reduces energy consumption by 21.87% compared to AODV, while Secure AODV reduces it by 43.92%. From the table, we can observe that the proposed Secure AODV algorithm consistently outperforms the AODV and Trust AODV calculations regarding throughput across all the quantity of hubs considered in the simulation. Specifically, for all the number of nodes, Secure AODV achieves higher throughput values than the other algorithms. For example, when there are 100 nodes, AODV achieves a throughput of 105 Mbps, Trust AODV achieves 150 Mbps, and Secure AODV achieves 175 Mbps. This represents a 66.7% increase in throughput compared to AODV and a 16.7% increase compared to Trust AODV. Overall, the results suggest that the throughput performance of the proposed Secure AODV algorithm is superior to that of the existing AODV and Trust AODV algorithms. The proposed and existing algorithms' throughputs are compared across varying numbers of nodes in Fig. 6.

**Figure 7:**
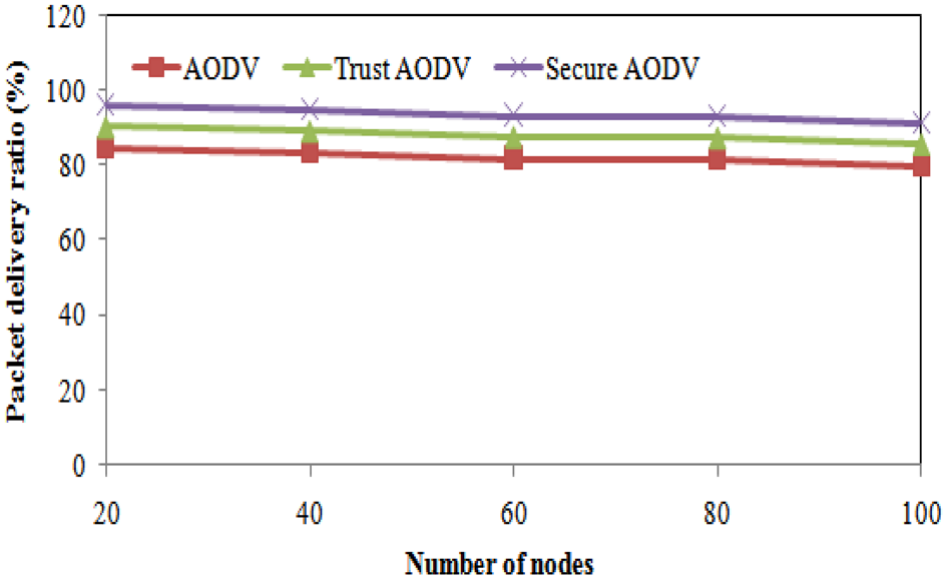
Packet delivery ratio comparisons with number of nodes varying.

**Figure 8:**
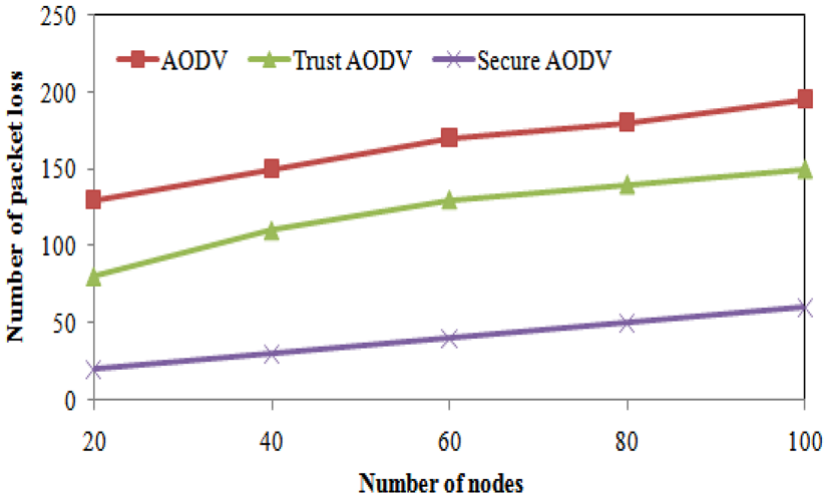
Number of packet loss comparisons with number of nodes varying.

**Figure 9:**
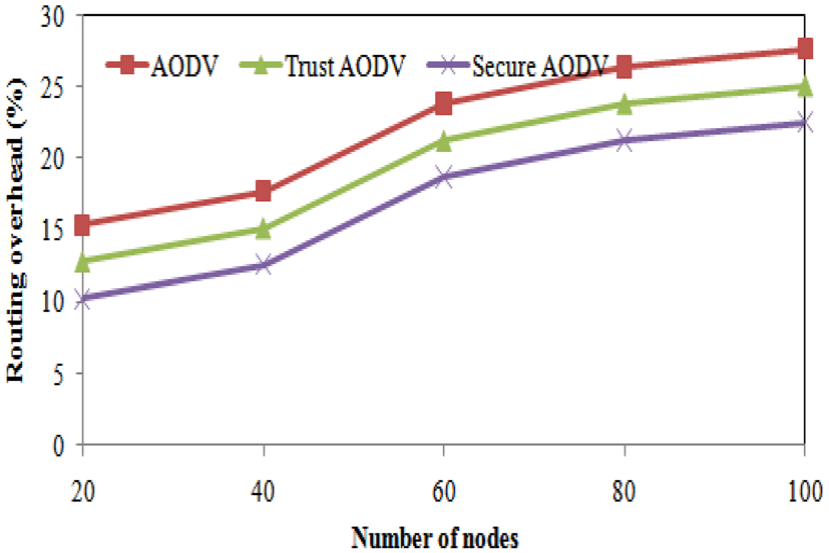
Routing overhead comparisons with number of nodes varying.

The Table 3 shows the comparison of packet delivery ratio (%) for AODV, Trust AODV, and Secure AODV with varying number of nodes. It can be observed that the proposed Trust AODV and Secure AODV protocols outperform the AODV protocol in terms of packet delivery ratio, as they achieve higher percentages across all numbers of nodes. The Trust AODV protocol achieves an increase of approximately 5–6% in packet delivery ratio compared to AODV, while Secure AODV achieves an increase of approximately 10–12%. These results indicate that incorporating trust and security mechanisms in routing protocols can significantly improve the packet delivery ratio in FANETs. Figure 7 shows the packet delivery ratio comparison of proposed and existing algorithm over number of nodes varying. The Fig. 8 shows the number of packet loss comparisons with number of nodes varying. The Fig. 9 depicts the Routing overhead comparisons with number of nodes varying.

From the table, it can be observed that the number of packet losses decreases as we move from AODV to Trust AODV and Secure AODV. For instance, in the case of 20 nodes, AODV has the highest number of packet losses at 130, while Secure AODV has the least at 20. Similarly, for 100 nodes, AODV has the highest number of packet losses at 195, while Secure AODV has the least at 60. This shows that Trust AODV and Secure AODV are better at maintaining packet delivery and reducing the number of packet losses compared to AODV. Figure 8 shows the number of packet loss comparison of proposed and existing algorithm over number of nodes varying. As the number of nodes increases, the routing overhead for all three protocols (AODV, Trust AODV, Secure AODV) also increases. However, the proposed Trust AODV and Secure AODV protocols show a significant reduction in routing overhead compared to AODV. For example, with 20 nodes, Trust AODV and Secure AODV show a 16.82% and 33.29% decrease in routing overhead, respectively, compared to AODV. With 100 nodes, this reduction increases to 9.11% and 18.51%, respectively. The proposed and existing algorithms' routing overheads are contrasted in Fig. 9 for varying numbers of nodes.

**Figure 10:**
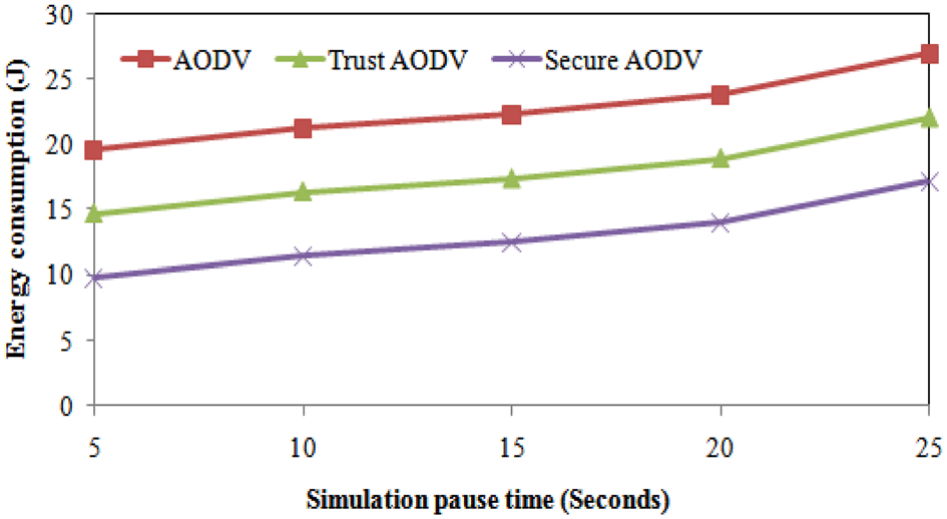
Energy consumption comparisons with simulation pause time.

Table 5 presents the comparison of the proposed Trust AODV and Secure AODV algorithms with the existing AODV algorithm in terms of the simulation pause time. To analyze the results with respect to simulation pause time, we have presented the energy consumption of AODV, Trust energy consumption for Trust AODV is 25.06% for 5 s, 23.05% for 10 s, 22.16% for 15 s, 20.37% for 20 s, and 18.19% for 25 s. Similarly, the percentage decrease in energy consumption for Secure AODV is 49.95% for 5 s, 38.92% for 10 s, 35.65% for 15 s, 29.31% for 20 s, and 17.41% for 25 s. These results suggest that the proposed Trust AODV and Secure AODV routing protocols are more energy efficient than AODV routing protocol. The comparison of the proposed and existing algorithms' energy consumption during the simulation pause time is depicted in Fig. 10. For AODV, the throughput increases as the simulation pause time increases. However, for Trust AODV and Secure AODV, the throughput increases up to 15 s and then starts decreasing as the simulation pause time increases further. This is because as the simulation pause time increases, the nodes stay idle for longer periods, which results in decreased network activity and hence reduced throughput. Trust AODV and Secure AODV have higher throughput compared to AODV for all values of simulation pause time.

**Table 5:** Results comparison of proposed and existing algorithms with respect to simulation pause time.

Simulation pauses time (seconds)	Energy consumption (J)			Throughput (Mbps)			Packet delivery ratio (%)		
	AODV	Trust AODV	Secure AODV	AODV	Trust AODV	Secure AODV	AODV	Trust AODV	Secure AODV
5	19.598	14.701	9.804	48	68	128	82.150	87.839	93.528
10	21.257	16.360	11.463	58	78	133	81.042	86.731	92.420
15	22.257	17.360	12.463	68	98	138	79.521	85.210	90.899
20	23.820	18.923	14.026	78	128	153	79.298	84.987	90.676
25	26.929	22.032	17.135	93	138	163	77.521	83.210	88.899

Simulation pauses time (seconds)	Number of packet loss			Routing overhead (%)					
	AODV	Trust AODV	Secure AODV	AODV	Trust AODV	Secure AODV			
5	115	65	5	18.932	16.364	13.796			
10	135	95	15	21.264	18.696	16.128			
15	155	115	25	27.354	24.786	22.218			
20	165	125	35	29.931	27.363	24.795			
25	180	135	45	31.176	28.608	26.040

**Figure 11:**
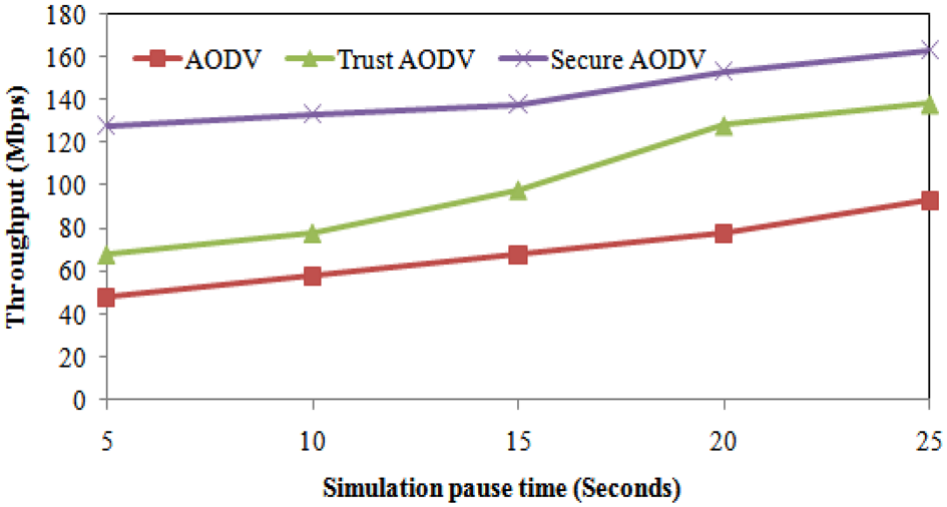
Throughput comparisons with simulation pause time.

The increase in throughput from AODV to Trust AODV is higher for smaller values of simulation pause time, while the increase from Trust AODV to Secure AODV is higher for larger values of simulation pause time. Overall, Secure AODV has the highest throughput for all values of simulation pause time. Trust AODV and Secure AODV show significant improvements in throughput compared to AODV, with Trust AODV showing a 41.67% to 82.35% increase and Secure AODV showing a 38.75% to 26.56% increase, depending on the simulation pause time. Figure 11 shows the throughput comparison of proposed and existing algorithm over simulation pause time.

**Figure 12:**
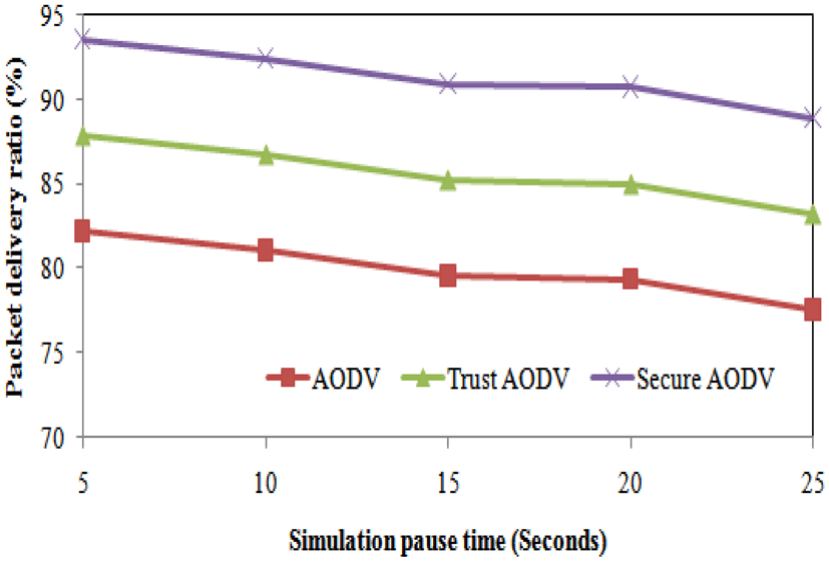
Packet delivery ratio comparisons with simulation pause time.

For the packet delivery ratio, we can see that Trust AODV and Secure AODV have a higher percentage of successful packet delivery compared to AODV for all simulation pause times. However, there is a slight decrease in the percentage for all algorithms as the simulation pause time increases. Trust AODV has the highest packet delivery ratio, with a maximum of 88% for a pause time of 5 s. In terms of percentage increase and decrease, we can see that the packet delivery ratio for Trust AODV and Secure AODV is higher than that of AODV for all simulation pause times. Figure 12 shows the packet delivery ratio comparison of proposed and existing algorithm over simulation pause time.

**Figure 13:**
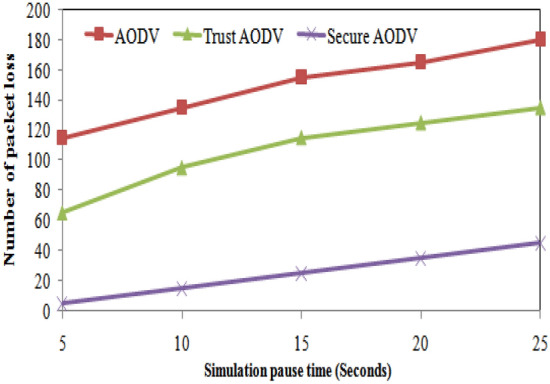
Number of packet loss comparisons with simulation pause time.

**Figure 14:**
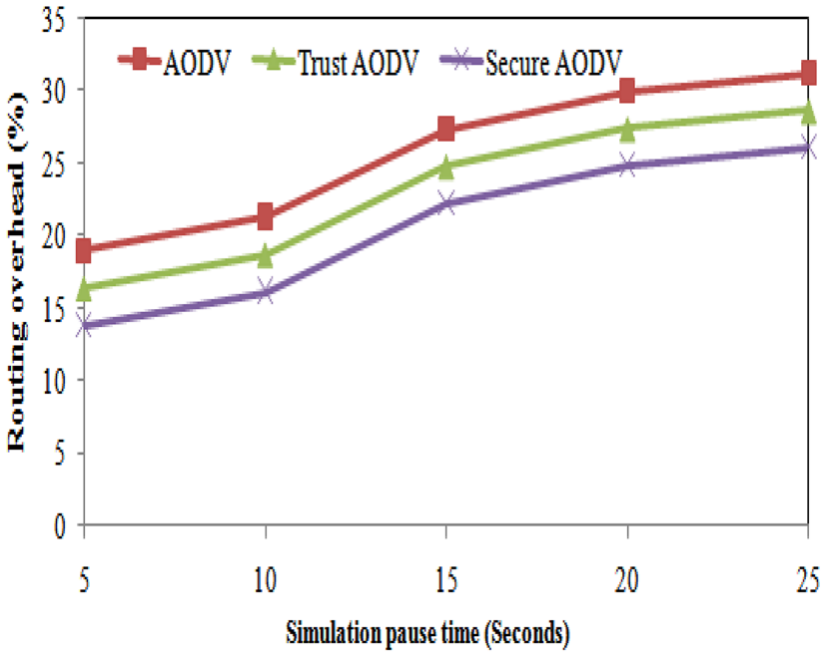
Routing overhead comparisons with simulation pause time.

The table shows the number of packet losses for different simulation pause times and for each of the three routing algorithms: AODV, Trust AODV, and Secure AODV. As the simulation pause time increases, the number of packet losses also increases for all three algorithms. This is expected because a longer pause time allows more time for potential disturbances or interference to occur. Comparing the three algorithms, Trust AODV and Secure AODV consistently have fewer packet losses than AODV for all simulation pause times. Figure 13 shows the number of packet loss comparison of proposed and existing algorithm over simulation pause time. As the pause time increases, the routing overhead for all three protocols also increases. However, the percentage increase is higher for Secure AODV and Trust AODV compared to AODV. For example, when the pause time increases from 5 to 25 s, the routing overhead for AODV increases by 65.6%, while for Secure AODV and Trust AODV, it increases by 85.6% and 78.1%, respectively. Figure 14 shows the routing overhead comparison of proposed and existing algorithm over simulation pause time.

## Comparative analysis with respect to state-of-art algorithms

The Table 6 compares the energy consumption (in J) of various algorithms including TBCS, HIDS-BS, WOA-OLSR, SEEDRP, AODV, RARP, RLPR, and the proposed algorithm Secure AODV, in relation to the number of nodes, which is 100. The proposed algorithm (Secure AODV) has the lowest energy consumption of all the algorithms compared, as shown in the table. The consumption of energy decreases from TBCS to RLPR and then to Secure AODV. This implies that the proposed algorithm is more energy-efficient compared to the existing state-of-the-art algorithms. Additionally, it can be seen that the difference between the energy consumption of Secure AODV and RLPR is higher than that between RLPR and RARP, indicating that the proposed algorithm has a significant improvement in energy efficiency compared to RLPR. Secure AODV shows a 58.87% decrease in energy consumption compared to TBCS. Secure AODV shows a 55.06% decrease in energy consumption compared to HIDS-BS. Secure AODV shows a 50.88% decrease in energy consumption compared to WOA-OLSR. Secure AODV shows a 45.08% decrease in energy consumption compared to SEEDRP. Secure AODV shows a 38.11% decrease in energy consumption compared to AODV. Secure AODV shows a 28.10% decrease in energy consumption compared to RARP. Secure AODV shows a 16.90% decrease in energy consumption compared to RLPR. In terms of throughput, Secure AODV achieved the highest value of 175 Mbps, which is again a significant improvement over the next best algorithm RLPR, which had a throughput of 163 Mbps. This represents an increase of 7.36% in throughput compared to RLPR. However, it is worth noting that the difference in throughput between the proposed algorithm and some of the other algorithms, such as AODV and RARP, is not very significant.

**Table 6:** Comparative analysis of proposed algorithm with existing state-of-art algorithms with respect to number of nodes 100.

References	Algorithm	Energy consumption (J)	Throughput (Mbps)	Packet delivery ratio (%)	Number of packet loss	Routing overhead (%)
^21^	TBCS	30.543	91	74.680	102	39.685
^22^	HIDS-BS	27.975	103	77.045	96	37.227
^26^	WOA-OLSR	25.407	115	79.410	90	34.769
^27^	SEEDRP	22.839	127	81.775	84	32.311
^28^	AODV	20.271	139	84.140	78	29.853
^28^	RARP	17.703	151	86.505	72	27.396
^28^	RLPR	15.135	163	88.870	66	24.938
Proposed model	Secure AODV	12.567	175	91.235	60	22.480

Among the considered algorithms, our Secure AODV outperforms all other algorithms with a packet delivery ratio of 91.235%. Specifically, the packet delivery ratios of the reference algorithms TBCS, HIDS-BS, WOA-OLSR, SEEDRP, AODV, RARP, and RLPR were 74.680%, 77.045%, 79.410%, 81.775%, 84.140%, 86.505%, and 88.870%, respectively. Therefore, it can be observed that our proposed Secure AODV algorithm has the highest packet delivery ratio among all algorithms. In terms of the percentage increase and decrease, the Secure AODV algorithm outperforms TBCS, HIDS-BS, WOA-OLSR, and SEEDRP by 22.55%, 18.48%, 11.83%, and 6.46%, respectively. It also outperforms AODV, RARP, and RLPR algorithms by 7.09%, 4.73%, and 2.36%, respectively. The superior performance of the Secure AODV algorithm can be attributed to its ability to ensure secure and reliable routing by employing trust management and cryptographic techniques. As the table indicates, the TBCS algorithm has the highest number of packet loss with 102 packets lost, while the proposed Secure AODV has the lowest number of packet loss with only 60 packets lost. In terms of percentage, the Secure AODV algorithm has a 23.08% decrease in packet loss compared to AODV, the conventional routing algorithm. Moreover, it has a 16.67% decrease compared to the RLPR algorithm, which has the lowest packet loss among the existing algorithms. The performance improvement in terms of packet loss is due to the enhanced security features and trust-based routing mechanism of Secure AODV that prevent packet loss caused by malicious nodes and congestion in the network. The routing overhead is measured in terms of the percentage of total transmitted packets used for routing control packets. The results show that TBCS has the highest routing overhead, with a value of 39.685%. HIDS-BS and WOA-OLSR have routing overheads of 37.227% and 34.769%, respectively. SEEDRP has a routing overhead of 32.311%, while AODV, RARP, and RLPR have routing overheads of 29.853%, 27.396%, and 24.938%, respectively. Our proposed algorithm, Secure AODV, has the lowest routing overhead of 22.480%. Compared to TBCS, Secure AODV reduces routing overhead by 43.3%, indicating that Secure AODV requires fewer control packets to be transmitted for routing. Compared to HIDS-BS, the routing overhead is reduced by 39.5%, while compared to WOA-OLSR, the reduction is 35.3%. Secure AODV also outperforms SEEDRP, which has a reduction in routing overhead of 30.6%, and AODV, which has a reduction of 24.4%. RARP and RLPR have higher routing overheads than Secure AODV, with reductions of 17.1% and 9.6%, respectively.

While simulations provide a controlled environment for assessing the performance of the proposed secure AODV algorithm, they may not fully capture the complexities and nuances of real-world FANET deployments. Factors such as environmental conditions, hardware constraints, and dynamic network behavior may not be accurately represented in simulations, leading to potential discrepancies between simulation results and real-world performance. Another limitation is the assumption of uniform node behavior and network conditions. The proposed algorithm may perform differently in scenarios where nodes exhibit varying levels of trustworthiness or operate under different environmental conditions. Incorporating heterogeneous node behavior and dynamic network conditions into the evaluation could provide a more comprehensive understanding of the algorithm's performance in diverse FANET environments. Additionally, the study may lack scalability analysis, particularly concerning large-scale FANET deployments. Evaluating the algorithm's performance in scenarios with a higher number of nodes and increased network complexity could uncover scalability issues or performance bottlenecks that are not evident in smaller-scale simulations. Furthermore, the study may not address all possible security threats and attack scenarios in FANETs. While the proposed algorithm aims to detect and isolate malicious nodes, it may overlook certain types of attacks or vulnerabilities that could compromise network security. Future research could explore additional security mechanisms or enhancements to address a broader range of security threats in FANETs.

## Conclusion

FANET is a distributed network that allows drone units to enter and exit the network as necessary. The routing, the quality of the service, and the security of FANET are three distinct issues. This paper discussed the malicious nodes attack in FANET and provides security by the proposed Secure AODV algorithm. A distributed denial of service attack known as a Sybil attack can flood a victim's host with raw data packets. In this research, we provide a mutual authentication mechanism to detect unauthorized nodes in the network. The proposed model will be implemented in NS2 to analyze that compared to AODV and TAODV, the FANET security architecture is better. The proposed secure AODV technique has maximum packets are received as 150 packets at 60 nodes in 8 s and the maximum of 390 packets are received at 60 nodes in 12 s. The proposed secure AODV technique has less packet loss as only 30 packets lost at 60 nodes in 8 s and the minimum of 20 packets are lost at 60 nodes. The proposed method received additional packets, in which only 50 packets were lost at 50 nodes in 8 s and a minimum of 20 packets, were received at 50 nodes in 12 s. The proposed method also reduces routing overhead and packet loss. In future, we contribute to the development of standardized protocols, architectures, and regulations specific to FANETs. Collaborate with industry stakeholders, regulatory bodies, and standardization organizations to ensure the safe and secure deployment of FANETs in real-world scenarios. One notable challenge is the scalability of the method. In larger FANETs with a substantial number of nodes, the computational and communication overhead for detecting and isolating malicious nodes may increase significantly. This could potentially lead to performance bottlenecks and necessitate more efficient algorithms for large-scale deployment. Implementing resource-intensive security algorithms on resource-constrained nodes may impact the overall system performance and efficiency. Like any intrusion detection system, there's the potential for false positives and false negatives. Misclassifying benign nodes as malicious (false positives) or failing to detect actual malicious nodes (false negatives) can impact the network's reliability and usability. Addressing these limitations and challenges will be critical to the successful real-world implementation of our proposed method.

## Data Availability

The datasets used and/or analysed during the current study available from the corresponding author on reasonable request.
